# Molecular Evidence of Coinfection with Acute Respiratory Viruses and High Prevalence of SARS-CoV-2 among Patients Presenting Flu-Like Illness in Bukavu City, Democratic Republic of Congo

**DOI:** 10.1155/2022/1553266

**Published:** 2022-04-09

**Authors:** Patrick Bisimwa Ntagereka, Rodrigue Ayagirwe Basengere, Tshass Chasinga Baharanyi, Théophile Mitima Kashosi, Jean-Paul Chikwanine Buhendwa, Parvine Basimane Bisimwa, Aline Byabene Kusinza, Yannick Mugumaarhahama, Dieudonne Wasso Shukuru, Simon Baenyi Patrick, Ronald Tonui, Ahadi Bwihangane Birindwa, Denis Mukwege

**Affiliations:** ^1^Molecular Biology Laboratory, Université Evangélique en Afrique, Bukavu, Congo; ^2^Faculty of Medicine, Université Evangélique en Afrique, Bukavu, Congo; ^3^Pathological Anatomy, Panzi General Referral Hospital, Bukavu, Congo; ^4^Department of Infectiology, Panzi General Referral Hospital, Bukavu, Congo; ^5^Institute of Basic Sciences, Technology and Innovation, Department of Molecular Biology and Biotechnology, Pan African University, Nairobi, Kenya; ^6^Gynaecology and General Surgery, Panzi General Referral Hospital, Bukavu, Congo

## Abstract

The coronavirus disease 2019 (COVID-19) is caused by severe acute respiratory syndrome coronavirus 2 (SARS-CoV-2), with clinical manifestation cases that are almost similar to those of common respiratory viral infections. This study determined the prevalence of SARS-CoV-2 and other acute respiratory viruses among patients with flu-like symptoms in Bukavu city, Democratic Republic of Congo. We screened 1352 individuals with flu-like illnesses seeking treatment in 10 health facilities. Nasopharyngeal swab specimens were collected to detect SARS-CoV-2 using real-time reverse transcription-polymerase chain reaction (RT-PCR), and 10 common respiratory viruses were detected by multiplex reverse transcription-polymerase chain reaction assay. Overall, 13.9% (188/1352) of patients were confirmed positive for SARS-CoV-2. Influenza A 5.6% (56/1352) and Influenza B 0.9% (12/1352) were the most common respiratory viruses detected. Overall, more than two cases of the other acute respiratory viruses were detected. Frequently observed symptoms associated with SARS-CoV-2 positivity were shivering (47.8%; OR = 1.8; CI: 0.88–1.35), cough (89.6%; OR = 6.5, CI: 2.16–28.2), and myalgia and dizziness (59.7%; OR = 2.7; CI: 1.36–5.85). Moreover, coinfection was observed in 12 (11.5%) specimens. SARS-CoV-2 and influenza A were the most cooccurring infections, accounting for 33.3% of all positive cases. This study demonstrates cases of COVID-19 infections cooccurring with other acute respiratory infections in Bukavu city during the ongoing outbreak of COVID-19. Therefore, testing for respiratory viruses should be performed in all patients with flu-like symptoms for effective surveillance of the transmission patterns in the COVID-19 affected areas for optimal treatment and effective disease management.

## 1. Introduction

The world is facing a pandemic caused by the coronavirus disease 2019 (COVID-19), with over 136 million confirmed cases, including approximately 2,941,128 deaths globally as of April 13, 2021 [1]. COVID-19 is caused by a severe acute respiratory syndrome coronavirus 2.

SARS-CoV-2 is a novel coronavirus that emerged in Wuhan, China, in December 2019 [2]. SARS-CoV-2 infection is generally asymptomatic [3], although in most confirmed cases, it may manifest itself with nontypical symptoms, including fever, cough, and sore throat [3–5], which are known to be present in a common cold. Additionally, anosmia and ageusia may be observed [6, 7].

The Democratic Republic of the Congo (DRC), with an estimated population of 86 million, reported the first COVID-19 case on March 10, 2020, in Kinshasa city. While in Bukavu, a city whose population is estimated above 1.13 million, registered the first case of COVID-19 from arriving travelers in March 2020. Due to delays in quarantine measures and the failure in applying ministry of health guidelines for containment of the pandemic, DRC had recorded 55774 confirmed cases and 1061 deaths as of 4^th^ September 2021 in all 26 provinces, including South Kivu.

South Kivu is characterized by a high population density, poor health infrastructure, and limited access to COVID-19 diagnostic tests. This places South Kivu among the high-risk provinces in the DRC. Previous studies have reported high seroprevalence of SARS-CoV-2 among health workers and travelers in Bukavu city, suggesting a high circulation of SARS-CoV-2 within this city [8, 9].

Diagnostic facilities for viral infections in DRC's provinces, and in particular in South Kivu, are scarce. This prevents systematic screening of individuals who are asymptomatic or present mild symptoms of COVID-19 (https://ourworldindata.org/coronavirus-testing). This challenge further hampers strategies in the evaluation of the spread of SARS-CoV-2 within the population. Currently, the COVID-19 “gold” test, which entails reverse transcription-polymerase chain reaction (RT-PCR) as recommended by the World Health Organization [3, 10], focuses on symptomatic cases, and hence, an accurate estimate of the incidence cannot be obtained.

Research on the clinical characteristics of COVID-19 in most African countries has shown that fever and cough are the most common symptoms associated with COVID-19 diagnosis. Aside from these signs, fatigue, difficulty in breathing, sore throat, headache, and other atypical symptoms have been reported.

The glaring similarity between these clinical signs with those of other respiratory viral infections [11] reduces the diagnostic efficiency and treatment of COVID-19 cases. This may lead to regular epidemics with pneumonia and bronchitis cases, severe respiratory failure, and even death [12].

Several studies have been carried out in different parts of the world on COVID-19 coinfection with other respiratory pathogens. Still, most of them were focused mainly on coinfection with the influenza virus A/B.

Unfortunately, in the DRC, no information on SARS-CoV-2 coinfection with other respiratory pathogens at the time this study was carried out is available. In addition, the persistence of flu-like symptoms has been observed frequently in SARS-CoV-2 patients after recovery. This led to increased hospitalization, adjusted treatment, and increased fatalities. The observation suggests possible coinfection of SARS-CoV-2 and other respiratory pathogens. Therefore, identifying SARS-CoV-2 and pathogens responsible for respiratory infections is critical in the development of strategies for better prevention and management of COVID-19 and other respiratory infections control. This could improve patient management and reduce patient isolation duration, particularly for those infected with other common respiratory viruses.

Thus, this study investigated the prevalence of SARS-CoV-2, influenza A and B, and other acute respiratory viruses among local patients with flu-like symptoms. These include but are not limited to patients presenting a sudden onset of a fever of >38°C and a cough or sore throat who were seeking treatment at different health structures in Bukavu city.

## 2. Methods

### 2.1. Study Area

This study was carried out in Bukavu, the capital city of South Kivu province in eastern DRC. The city lies at the extreme south-western edge of Lake Kivu, separated from Rwanda by the outlet of the Ruzizi River. As of 2020, Bukavu had an estimated urban population of 1,133,371 (https://populationstat.com/democratic-republic-of-the-congo/bukavu). Moreover, the city is characterized by a tropical wet and dry climate with 1498 m of altitude above sea level, an average rainfall of about 1224 mm annually, and is located between 2.5123° S and 28.8480° E. Patients were recruited from December 2020 to May 2021 in 10 health facilities such as BIOPHARM Health Centre, Panzi General Reference Hospital, Saint Vincent polyclinic, MUHUNGU Health Centre, DIOCESAIN Centre, Saint Luc polyclinic, 5^th^ CELPA Health Centre, SOS Medical Centre, Nyantende Hospital, and CHAI Health Centre.

### 2.2. Study Design, Participants, and Sample Collection

A cross-sectional study was adopted. Collection of swab samples to confirm COVID-19 disease among patients with flu-like symptoms defined as an outpatient with a sudden onset of a fever >38°C, a cough, or sore throat seeking treatment at different health facilities. Only individuals with flu-like disease symptoms, aged ten years and above, and able to give consent were considered for sample collection during the interview. Personal data such as age, sex, occupation, and residence were recorded prior to collecting oropharyngeal swab samples. Collected samples were put in a 2 ml tube containing 2 milliliters of Sample Storage Reagent (Ansure, Biotechnology) composed of 0.9% of normal saline raisin and stored at −40°C before SARS-CoV-2 Reverse Transcriptase Polymerase Chain Reaction amplification.

A total of 1352 Oropharyngeal samples were collected and shipped daily in ice to the Molecular Biology laboratory of the Université Evangélique en Afrique (UEA). The data collection followed ethical guidelines applied for human biological data collection and processing during the study.

### 2.3. Molecular Detection of SARS-CoV-2

Viral RNA was extracted from swab samples within 1–24 hours following sample collection using the innuPREP Virus TS RNA kit (Analytik Jena, Berlin, Germany) according to the manufacturer's instructions. The real-time reverse transcriptase-polymerase chain reaction (RT-PCR) tests for SARS-CoV-2 were performed using the TIB MOLBIOL Sarbeco E-gene Plus EAV PCR Kit (Olfert Landt, Berlin, Germany). Briefly, this entailed a 20 *μ*L reaction mix comprising 10 *μ*L of 2x Reverse Transcriptase Master Mix, 0.5 *μ*L of extraction control EAV, 0.5 *μ*L of PSR reagent (SARS-CoV-2 probes and primers mix targeting the viral *E*-gene), 5 *μ*L of the extracted RNA, and the volume was adjusted to 20 *μ*L with nuclease free water.

The cDNA synthesis step was conducted by incubating the mixture at 55°C for 5 minutes. RT-PCR reactions started with an initial denaturation step at 95°C for 5 min, followed by cycling steps of 95°C for 5 seconds, primer annealing at 60°C for 15 seconds, and an extension step of 72°C for 15 seconds. All the RT-PCR reactions were carried out in the LightCycler® 96 Instrument (Roche Life Science). A sample was considered positive if the threshold (Ct value) was <35, while any sample with a threshold (Ct value) >35 was considered as negative.

### 2.4. Detection of Common Acute Respiratory Viruses by Multiplex RT-PCR Assays

Each RNA sample was subjected to multiplex RT-PCR amplification using the ARVI Screen Real-TM Multiplex Detection kit (Sacace Biotechnologies, Italy) on a LightCycler 96 detection system (Roche, Germany). The panel was specifically developed to identify ten viruses including human respiratory syncytial virus (hRSV); human metapneumovirus (hMpv); human parainfluenza virus-1-4 (hPiv-1-4), ОС43, Е229, NL63, and HKUI human coronavirus (hCov); human rhinovirus (hRv); human B, C, and E adenovirus (hAdv), human bocavirus (hBov), as well as influenza A and B within 1 hour. The assay comprised reverse transcription and multiplex RT-PCR amplification.

The cDNA synthesis was performed in a 40 *μ*L mix containing 5 *μ*l RT-G-mix-1, 6 *μ*l of reverse transcriptase (M-MLV), 9 *μ*L of RT-Mix, and 20 *μ*L of RNA as a template. The cDNA libraries were constructed by incubating the reaction mix at 37°C for 30 minutes in a PeQlab thermocycler (peqSTAR, VWR). The cDNA samples were diluted in half with T. E. buffer for immediate use in multiplex RT-PCR amplification or storage at −20°C for future analysis.

In addition, the multiplex real-time amplification setup was carried out. Each mix contained primers directed against regions of acute respiratory viral infection (ARVI). For each mix, three controls were also included, such as negative, positive, and internal control.

Real-time RT-PCR assays were performed in a 25 *μ*L reaction comprising 10 *μ*L of PCR-mix 1, containing specific primers for each virus, 5 *μ*L of PCR-Mix-FRT and 10 *μ*L of template cDNA. The cycling parameters entailed 95°C initial denaturation for 15 min, followed by 40 cycles of 95°C denaturation for 10 s, 54°C annealing for 25 s, and extension at 72°C for 25 s. We set our threshold value for positive cases of ARVI pathogens at any value less than 35.

The negative cases of an ARVI were assessed when the threshold value (Ct) of the tested sample was absent (not determined) or if the Ct value exceeded 35.

### 2.5. Data Analysis

The collected data were encoded in Microsoft® Excel® data management tool combining the results of SARS-CoV-2 RT-PCR and other respiratory virus assays and data obtained from the questionnaire. Prior to statistical analyses, all personal identification data were eliminated. Descriptive statistics were calculated for all of the variables, including the RT-PCR prevalence results. We assessed the associations between SARS-CoV-2 and other respiratory virus RT-PCR results (positive or negative) with age, sex, symptom, exposure, and morbidity using the Chi-squared test. The odds of being a positive case based on RT-PCR results were then modeled as a function of the dichotomous risk factors measures, using logistic regression models. All variables with *p* values of ≤0.05 were considered statistically significant. The R Console statistic software (version 4.0.0) was used to perform the analysis.

**Figure 1:**
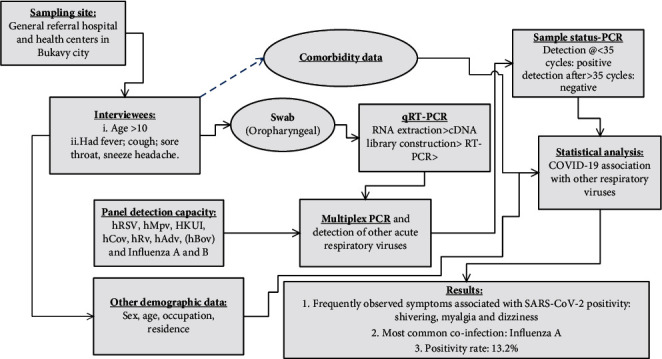
Study design, participants, and results of COVID-19 and other acute respiratory viruses coinfection.

Figure 1 represents the flowchart/schematic that describes the study design, participants, and results of COVID-19 and other acute respiratory viruses coinfection and meta-analysis.

## 3. Results

### 3.1. SARS-CoV-2 and Common Acute Respiratory Infection Prevalence and Associated Symptoms in Patients with Flu-Like Symptoms from Nine Health Structures

In total, 1352 individuals presenting flu-like illness were examined, all of whom resided in Bukavu city. Among these were 696 males (51.4%) and 656 females (48.5%). The majority (89.6%) were adults. A high proportion (63.3%) were those recorded in the BIOPHARM medical center. The SARS-CoV-2 was detected in 188, representing a positivity rate of 13.9%. The prevalence of SARS-CoV-2 was higher in females (14.6%) compared to males (13.3%). The age range of the SARS-CoV-2-positive patients was 176 (14.5%) out of 1212 adults and 12 (8.6%) of the young (less than 18 years) out of 140.

Influenza A and B viruses were diagnosed in 76 (5.6%) and 12 (0.9%) samples. The multiplex RT-PCR test detected human parainfluenza 1–4 (hPiV 1–4) in 10 (0.7%) patients, while human metapneumonia virus (hMpv) was present in 5 (0.4%) patients. Additionally, human coronavirus (hCoV), human rhinovirus (hRV) and human adenovirus (hAdV) were identified in 3 (0.2%) samples for each, while only 1 (0.07%) patient was identified to be infected with human bocovirus (hBoV).

Of all the health facilities, Panzi General Reference (Panzi HGRP) recorded the highest SARS-CoV-2, 12 (23.1%), and influenza B, 4 (7.7%) cases (Table 1).

### 3.2. Profiles of SARS-CoV-2 and Other Respiratory Infection Positive Patients and Associated Clinical Symptoms

The most frequent symptom in COVID-19 was coughing (89.6%), followed by headaches (1049, 77.6%), asthenia (909, 67.2%), high fever (706, 52.2%), as well as myalgia (59.7%). Hypertension (122, 9%), obesity (81, 6%), and diabetes (81, 6%) were the most frequently reported comorbidities in COVID-19 positive individuals. More than half of influenza A-positive patients experienced fever, cough, and headache and were asthenia, hypertensive, and diabetics. However, all influenza B-infected patients experienced headaches (100%) while more than half presented fever, coldness, cough, breathing difficulties, asthenia, myalgia, anorexia, and sputum (Supplementary materials Table 1**).** The statistical analysis revealed statistically significant association between COVID-19 with some symptoms such as shivering (47%. 8%; OR = 1.8; CI: 0.88–1.35; *p*=0.024), cough (89.6%; OR = 6.5, CI = 2.16–28.2; *p*=0.003), myalgia, and dizziness (59.7%; OR = 2.7; CI: 1.36–5.85, *p*=0.006) (Table 2).

A comparative analysis between SARS-CoV-2 positive and negative individuals showed that patients who had been in contact with infected persons presented a higher risk of being infected by SARS-CoV-2 (OR = 2.40; CI: 0.43–13.45), followed by workers in hospitals (OR = 1.78; CI: 0.34–8.43) and patients with diabetes (OR = 1.17; CI: 0.29–3.93), than negative individuals. However, none of these factors was statistically significant. Additionally, no statistical difference was found between gender and commodities variables when comparing SARS-CoV-2 and negative patients (*p* < 0.05) (Table 2).

### 3.3. Logistic Regression Analysis for Factors Associated with Influenza A and B Positivity

The logistic regression analysis revealed that 53.3% (OR = 1.51; IC = 0.46–4.98) of influenza A negative patients were inversely correlated with cold compared with 16.7% in influenza A positive patients. However, there were no significant differences in other factors for influenza A and B infection rates (*p* > 0.05) (Table 3).

### 3.4. Coinfection Patterns of COVID-19 and Other Acute Respiratory Viruses

During this investigation, six coinfection patterns were found. Out of the 113 cases of infected acute respiratory patients, 12 (11.5%) were co-infected with different acute respiratory viruses. A high frequency of coinfection pathogens involved SARS-CoV-2 and influenza A, accounting for 33.3% of all coinfection cases. Additionally, 3 cases (25%) of hMPiV positive cases were coinfected with hCoV, followed by 2 cases (16.6%) of SARS-CoV-2 coinfected with influenza B (Table 4).

## 4. Discussion

COVID-19's first reports in the DRC emerged towards the beginning of March 2020, and there has been an upsurge of cases since then nationally. SARS-CoV-2 infections in South Kivu province soared from April to December 2020. This is the period when flu-like illnesses peak. Therefore, it was to investigate the presence of SARS-CoV-2 among local patients with flu-like illnesses seeking treatment in various health facilities. This may help us control the epidemic better and handle other acute respiratory infections sufficiently. This study included 696 males (51.4%) and 656 females (48.5%), and the majority of those, 1212 (89.6%), were adults.

In this study, a total of 188 participants were COVID-19 positive, giving a positivity rate of 13.9%. This rate is higher than those reported in China (0.3%) and South Kivu (41.2%). [9]. The difference could probably be explained by the type of test and study population. The previous study from HGRP detected only antibodies (exposure) and focused only on asymptomatic health workers. In contrast, the current study examined individuals presenting flu-like illness seeking treatment in different health facilities.

In addition, 8.3% of the flu-like illness patients tested positive for other respiratory viruses. These results emphasize the importance of simultaneous diagnosis of other acute respiratory viral pathogens, particularly in a mild epidemic area and during the current COVID-19 pandemic.

Among the acute respiratory viral pathogens, influenza A and influenza B viruses were the most detected in 76 (5.6%) and 12 (0.9%) patients with flu-like symptoms and COVID-19 negative. This infection rate is relatively lower than the 40.4% influenza A and 14.3% influenza B reported in Shanghai, respectively [13]. In Chongqing, 65.7% cases of influenza A and 34.1% of influenza B were reported in individuals with flu-like symptoms [14]. This differences could be explained by the difference in targeted population, the current study was focused mostly on people aged ten years and above while in the previous studies the majority were children and young people. Differences in climatic conditions of the sampled regions could also contribute to different rates of infection. However, this study's findings agree with a recent study revealing a higher incidence of influenza infections in children and adolescents compared to old individuals [15].

Furthermore, other respiratory pathogens were detected including hPiV 1–4, hPiV 1–4 10 (0.7%), hMPiV 5 (0.4), and the other viruses with 3 cases (0.2%) each. Similar results were obtained in different studies in Costa Rica and China [16]. In all the cases, adults tended to be most affected compared to young people, although this was not statistically different.

A weak association was noted in patients infected with respiratory viruses and clinical signs such as cough, headaches, asthenia, feeling cold, fever, shivering, and myalgia. This result agrees with the finding from Monto et al. [17] that showed cough associated with fever to be the most frequent symptom of influenza infection. However, our findings are inconsistent with a previous study that focused on the clinical features of COVID-19. Influenza, which demonstrated that symptoms such as anosmia, dysgeusia, diarrhea, frontal headache, and bilateral cracklings sounds were more frequent in COVID-19 while sputum production, dyspnea, sore throat, conjunctivitis, hyperthermia, tearing, vomiting, and rhonchi sounds were more frequent in influenza infection [18]. A subsequent study with a larger sample and follow-up of patients would establish clinical signs of similarities and differences between infection with COVID-19 and infection with other respiratory viruses. This can help practically in the areas under-equipped for the biological diagnosis.

In this study, gender and comorbidities did not significantly influence the COVID-19 rate. This is similar to previous studies showing that gender was not associated with the SARS-CoV-2 infection rate [19, 20].

The risk of being infected by SARS-CoV-2 increased in patients who had been in contact with diseased persons (OR = 2.4). This is in accordance with the fact that in resource-limited areas, including South Kivu province, quarantine of infected individuals often occurs at home, enabling onwards transmission within households.

People of all ages are susceptible to SARS-CoV-2. However, previous findings have confirmed that adults, specifically older men, are more likely to be affected and subsequently suffer from severe pneumonia, pulmonary oedema, acute respiratory distress syndrome, multiple organ failure, and death [5, 21, 22]. Although it appeared that all population ages were susceptible to SARS-CoV-2 infection, the median age of infection was in adult persons (around 50 years). This is in agreement with previous research revealing the median age of infection to be around 50 years [19] and considering the range of the African population.

In general, there are no striking differences in the clinical behavior and severity between the different influenza A and B viruses affecting patients. The only significant clinical symptom with a negative relationship to influenza A virus was feeling cold (OR = 0.18, *p*=0.017). Our data do not show differences in clinical manifestation compared with other previous descriptions in the literature. However, four clinical symptoms were relevant for influenza A, including fever, cough, headache, and feeling cold. Similar results were reported in a previous study in Costa Rica [15]. Among the 12 patients who were influenza A positive, all (100%) experienced a headache, and more than half presented the remaining symptoms such as fever, coldness, shivering, sweating, cough, breathing difficulties, essoufflés, asthenia, myalgia, anorexia, sputum, and dyspnea. No respiratory and cardiac failure could be associated with influenza infection. This result is in contrast with previous studies that have reported 16% of cases of seasonal influenza occurring in clinical pulmonary patients [23] and 25.2% in cardiac failure [24].

Moreover, 12 (11.5%) cases coinfected with other acute respiratory viruses were reported. The highest coinfection pathogens were SARS-CoV-2 and influenza A, accounting for 33.3% of all coinfection cases. The coinfection rate between SARS-CoV-2 and influenza virus can probably be explained by the seasonality nature of the influenza virus which peaks between December and April, corresponding to the winter period. This observation is supported by a previous study conducted by Leung et al. [25]. However, our findings are in contrast with previous studies [26, 27] whose findings show a decrease in seasonal influenza cases during the COVID-19 pandemic. In this regard, it would be ideal to have the follow-up patients coinfected with COVID-19 and other respiratory viruses and assess their evolution over time; this may also require a separate study for clinical follow-up of cases.

Additionally, we recorded cases of hMPiV coinfected with hCoV. These findings are in accordance with previous studies suggesting the possible coinfections of multiple respiratory viruses [28–31].

This study demonstrates cases of COVID-19 infections co-occurring with other acute respiratory infections in the DR Congo. These data on the coinfection of SARS-CoV-2 with respiratory viral pathogens emphasizes the need for routine testing of multiple viral pathogens in mild epidemic areas during the current COVID-19 epidemic. Further study is needed to evaluate clinical patients with these coinfections and their evolution. Additionally, whether simultaneous viral infection in SARS-CoV-2 patients can potentially drive viral interference or impact disease outcome needs further investigation.

## 5. Conclusion

To our knowledge, this is the first report in DRC that demonstrates the co-occurrence of SARS-CoV-2 with other respiratory pathogens in the DRC. Current treatments and management strategies for flu-like presenting ailments focus on the diagnostics that are inclined to SARS-CoV-2 alone. Hence, our study reveals that there may be more underlying infections that call for combinatorial therapies for effective treatment and management of symptoms accompanying SARS-CoV-2 symptoms. As such, physicians should be considering that a positive test for COVID-19 does not exclude the possibility of other respiratory diseases.

## Figures and Tables

**Table 1:** Frequency of SARS-CoV-2 and other respiratory viruses detected according to sex, age, and health facility.

Variables	Number sampled	SARS-CoV-2 +	INFL. *A* +	INFL. *B* +	hMpv	hPiV 1–4	hCoV	hRV	hAdV	hBoV
Gender										
F	696	101 (14.6%)	40 (5.8%)	8 (1.2%)	3 (0.4%)	7 (1.0%)	1 (0.1%)	2 (0.3%)	1 (0.1%)	1 (0.1%)
M	656	87 (13.3%)	36 (5.5%)	4 (0.6%)	2 (0.3%)	3 (0.5%)	2 (0.3%)	1 (0.2%)	2 (0.3%)	0 (0%)
Age range										
Adult	1212	176 (14.5%)	60 (5.0%)	12 (1.0%)	4 (0.3%)	8 (0.7%)	3 (0.2%)	3 (0.2)	3 (0.2%)	1 (0.1%)
Young	140	12 (8.6%)	12 (8.6%)	0 (0%)	1 (0.7%)	2 (1.4%)	0 (0%)	0 (0%)	0 (0%)	0 (0%)
Health structures										
Biopharm HC	856	116 (13.6%)	48 (5.6%)	4 (0.5%)	1 (0.1%)	4 (0.5%)	0 (0%)	0 (0%)	0 (0%)	1 (0.1%)
5^th^ CELPA HC	72	8 (11.1%)	4 (5.6%)	0 (0%)	1 (1.4%)	2 (2.8%)	1 (1.4%)	1 (1.4%)	2 (2.8%)	0 (0%)
Panzi GRH	52	12 (23.1%)	0 (0%)	4 (7.7%)	2 (3.8%)	0 (0%)	2 (3.8%)	0 (0%)	0 (0%)	0 (0%)
CHAI HC	64	4 (6.3%)	4 (6.3%)	0 (0%)	0 (0)	0 (0%)	0 (0%)	0 (0%)	0 (0%)	0 (0%)
SOS HC	88	16 (18.2%)	4 (4.5%)	0 (0%)	0 (0%)	0 (0%)	0 (0%)	0 (0%)	0 (0%)	0 (0%)
Nyantede hospital	76	12 (15.8%)	4 (5.3%)	0 (0%)	0 (0%)	0 (0%)	0 (0%)	1 (1.3%)	0 (0%)	0 (0%)
Muhungu dios. HC	20	4 (20.0%)	0 (0%)	0 (0%)	0 (0%)	0 (0%)	0 (0%)	0 (0%)	0 (0%)	0 (0%)
St luc HC	24	8 (33.3%)	4 (16.7%)	0 (0%)	1 (4.2%)	2 (8.3%)	0 (0%)	1 (4.2%)	1 (4.2%)	0 (0%)
Muhungu HC	28	8 (2.9%)	4 (1.4%)	4 (1.4%)	0 (0%)	2 (0.7%)	0 (0%)	0 (0%)	0 (0%)	0 (0%)
Total N (%)	**1352**	**188 (13.9)**	**76 (5.6)**	**12 (0.9)**	**5 (0.4)**	**10 (0.7)**	**3 (0.2)**	**3 (0.2)**	**3 (0.2)**	**1 (0.07)**

**Table 2:** Association of flu-like symptoms with SARS-CoV-2 positivity among flu-like patients seeking treatment in different health facilities.

Variables	Negative covid19. *N* = 1164 (%)	Positive Covid19. *N* = 188 (%)	Or (CI)	*p* value
Symptoms (%)				
Fever	512 (44.1%)	98 (52.2%)	1.1 (0.560–2.282)	0.734
Coldness	531 (45.6%)	98 (52.2%)	0.9 (0.456–1.856)	0.816
Shivering	371 (31.9%)	88 (47.8%)	1.8 (0.88–1.35)	0.0246^*∗∗*^
Sweating	268 (23.0%)	56 (29.9%)	1.3 (0.603–3.69)	0.452
Cough	764 (65.6%)	168 (89.6%)	6.5 (2.161–28.207)	0.003^*∗∗∗*^
Breathing difficulties	271 (23.3%)	45 (23.9%)	0.7 (0.321–1.814)	0.590
Essoufflés	233 (20.0%)	17 (9.0%)	0.3 (0.107–1.094)	0.0992
Headaches	793 (68.1%)	146 (77.6%)	1.6 (0.738–3.821)	0.242
Asthenia	560 (56.7%)	126 (67.2%)	1.6 (0.804–3.487)	3.487
Myalgia	478 (41.1%)	112 (59.7%)	2.7 (1.363–5.859)	0.006^*∗∗∗*^
Anorexia	314 (27.0%)	73 (38.8%)	1.5 (0.743–3.283)	0.232
Sputum	194 (16.7%)	42 (22.4%)	0.8 (0.360–1.926)	0.716
Dyspnea	164 (14.1%)	19 (10.4%)	0.6 (0.236–1.772)	0.461
Expositions (%)				
Stay in high risk areas	116 (10.0)	33 (17.9)	1.09 (0.31–2.81)	0.85
Close contact	48 (4.1)	11 (6.0)	2.40 (0.43–13.45)	0.29
Work in hospitals	81 (7.0)	17 (9.0)	1.78 (0.34–8.43)	0.46
Comorbidities (%)				
HIV infection	8 (0.7)	0 (0.0)	—	—
Arterial hypertension	112 (9.6)	17 (9.0)	0.82 (0.21–2.57)	0.75
Obesity	30 (2.6)	11 (6.0)	0.76 (0.03–6.16)	0.81
Diabetes	69 (5.9)	11 (6.0)	1.17 (0.29–3.93)	0.80
Chronic respiratory dis.	26 (2.2)	3 (1.5)	0.56 (0.02–3.97)	0.61
Cardio pathology	8 (0.7)	0 (0.0)	—	—
Chronic kidney dis.	13 (1.1)	0 (0.0)	—	—
Nero pathology	5 (0.4)	1.5	0.00 (0.00–0.001)	0.98

^*∗∗∗*^Highly significant; ^*∗∗*^very significant; ^*∗*^significant.

**Table 3:** Logistic regression analysis regarding demographical gender, exposition, and comorbidity between positive influenza A and B patients.

	*INFL A*		INFL. *A*		INFL *B*		INFL. *B*	
	**Neg. (N = 1276)**	**Pos. (N = 76)**	**OR (IC)**	*p*	**Neg. (N = 1340)**	**Pos. (N = 12)**	**OR (IC)**	*p*
**Symptoms (%)**								
Fever	48.2	58.3	1.51 (0.46–4.98)	0.558	49.0	51.2	1.04 (0.21, 5.22)	1.000
Coldness feeling	53.3	16.7	0.18 (0.04–0.83)	0.017^*∗∗*^	50.3	51.5	0.99 (0.20, 4.94)	1.000
Shivering	40.1	25.0	0.50 (0.13–1.92)	0.369	38.8	51.5	1.58 (0.32, 7.91)	0.682
Sweating	24.8	25.0	1.01 (0.26–3.95)	1.000	25.2	0.0	—	—
Cough	77.4	58.3	0.41 (0.12–1.38)	0.163	76.2	51.3	0.31 (0.06, 1.57)	0.155
Breathing difficulties	22.6	25.0	1.14 (0.29–4.47)	1.000	22.4	51.2	3.45 (0.69, 17.38)	0.135
Essoufflés	16.1	25.0	1.74 (0.44–6.95)	0.425	17.0	0.0	—	—
Headaches	71.5	66.7	0.80 (0.23–2.80)	0.745	70.7	100.0	—	—
Asthenia	59.1	50.0	0.69 (0.21–2.25)	0.556	58.5	51.5	0.71 (0.14, 3.55)	0.697
Myalgia	48.9	25.0	0.35 (0.09–1.34)	0.139	46.9	51.5	1.13 (0.23, 5.66)	1.000
Anorexia	31.4	25.0	0.73 (0.19–2.83)	0.755	30.6	51.5	2.27 (0.45, 11.37)	0.379
Sputum	25.5	8.3	0.26 (0.03–2.13)	0.295	23.8	51.1	3.20 (0.64, 16.09)	0.155
Dyspnea	16.1	25.0	1.74 (0.44–6.95)	0.425	17.0	0.0	—	—
**Expositions (%)**								
Stay in high risk areas	14.6	16.7	1.17 (0.24–5.74)	0.692	15.0	0.0	—	—
Close contact	4.4	0.0	—	—	4.1	0.0	—	—
Work in hospitals	5.1	0.0	—	—	4.8	0.0	—	—
**Comorbidities (%)**								
Immunosuppression	0.7	0.0	—	—	0.7	0.0	—	—
Hypertension	9.5	16.7	1.91 (0.38–9.66)	0.346	10.2	0.0	—	—
Obesity	2.2	8.3	4.06 (0.39–42.37)	0.288	2.7	0.0	—	—
Diabetes	7.3	16.7	2.54 (0.49–13.21)	0.249	8.2	0.0	—	—
Chronic resp. Dis.	3.6	0.0	—	—	3.4	0.0	—	—
Chronic cardiac dis.	0.0	0.0	—	—	0.0	0.0	—	—
Chronic kidney dis.	0.7	0.0	—	—	0.7	0.0	—	—
Chronic liver dis.	0.0	0.0	—	—	0.0	0.0	—	—
Chronic neurologic dis.	0.7	0.0	—	—	0.7	0.0	—	—

^*∗∗*^Very significant.

**Table 4:** Coinfection of SARS-CoV-2 with others respiratory viruses in flu-like symptomatic patients.

Number	Virus types	Coinfectious viruses	Cases
1	hMPV + hPiV 1–4	2	1
2	hMPV + hCoV	2	3
3	HBoV + Inf. A	2	1
4	SARS-CoV-2 + Inf. B	2	2
5	SARS-CoV-2 + Inf. A	2	4
6	SARS-CoV-2 + hMPiV	2	1

## Data Availability

The clinical data used to support the findings of this study are available from the corresponding author upon request.

## Supplementary Materials

Supplementary Materials Supplementary material Table 1: characteristics of the overall flu-like SARS-CoV-2 positive patients and others respiratory viruses.
